# Supplementary material for: The Small RNA Universe of Capitella teleta
Source: Front Mol Biosci. 2022 Feb 25;9:802814. doi: 10.3389/fmolb.2022.802814 (PMC8915122; doi:10.3389/fmolb.2022.802814)
Supplement: Supplementary file 1 [file DataSheet1.ZIP › Supplement/known/CAPTEscaffold_296_17119.pdf]

| | | |
|---|---|---|
| Provisional ID | : | CAPTEscaffold_296_17119 |
| Score total | : | 72685.4 |
| Score for star read(s) | : | 3.9 |
| Score for read counts | : | 72674.7 |
| Score for mfe | : | 2.2 |
| Score for randfold | : | 1.6 |
| Score for cons. seed | : | 3 |
| Total read count | : | 142560 |
| Mature read count | : | 142441 |
| Loop read count | : | 0 |
| Star read count | : | 119 |

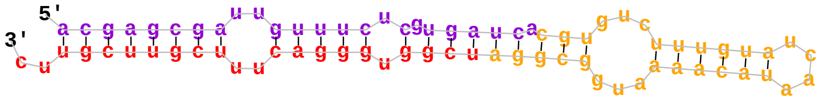


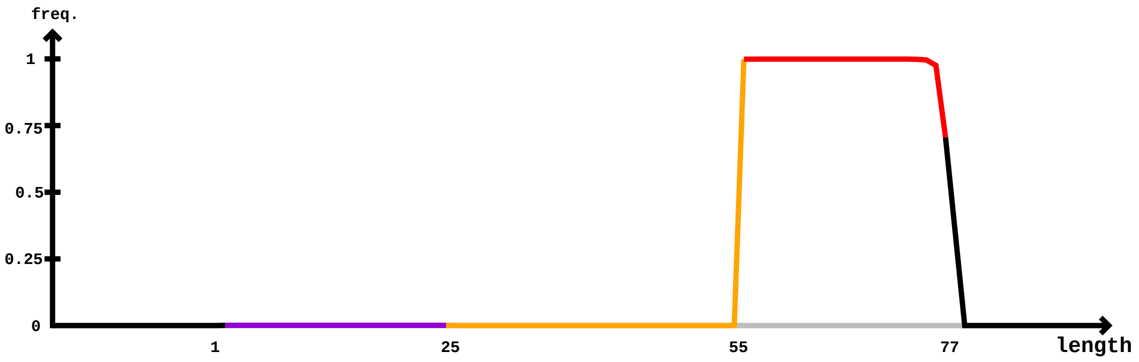


```
                 Star                                           Mature
5'- uguacgcccgucgcccacgagcgauuguuucucgugaucacgugucuuuguaucaauacaaaauggcggaucggugggacuuucguucguucggggcgggcggauuaa  -3'  obs
    uguacgcccgucgcccacgagcgauuguuucucgugaucacgugucuuuguaucaauacaaaauggcggaucggugggacuuucguucguucggggcgggcggauuaa       exp
    uguacgcccgucgcccacgagcgauuguuucucgugaucacgugucuuuguaucaauacaaaauggcggaucggugggacuuucguucguucggggcgggcggauuaa       known
    ....((((((((.(..((((((((..(((((.(.(((((.(((...((((((....))))))...))))))))).))))).))))))))..).))))))))......       reads  mm  sample
    ................acgagcgauuguuucucgugauc.....................................................................       21     0   seq
    ................acgagcgauuguuucucguAauc.....................................................................       1      1   seq
    ................acgagcgauuguuucucgugaucU....................................................................       97     1   seq
    .....................................................................Cucggugggacuuucguucgu.................       1      1   seq
    .....................................................................Cucggugggacuuucguucguu................       6      1   seq
    .....................................................................Cucggugggacuuucguucguuc...............       35     1   seq
    .....................................................................aucggugggacuuucguucguucA...............       3      1   seq
    ......................................................................ucggugggacuuucguuU....................       1      1   seq
    ......................................................................ucggugggCcuuucguuc....................       1      1   seq
    ......................................................................ucggugggacuuucguuc....................       122    0   seq
    ......................................................................ucggugAgacuuucguuc....................       1      1   seq
    ......................................................................ucgguggUacuuucguucg...................       1      1   seq
    ......................................................................ucggugggacuuuGguucg...................       1      1   seq
    ......................................................................Gcggugggacuuucguucg...................       2      1   seq
    ......................................................................ucggugggacuuucguucg...................       313    0   seq
    ......................................................................ucAgugggacuuucguucg...................       1      1   seq
    ......................................................................ucggugAgacuuucguucg...................       1      1   seq
    ......................................................................ucCgugggacuuucguucg...................       1      1   seq
    ......................................................................ucggugggacCuucguucg...................       1      1   seq
    ......................................................................Ncggugggacuuucguucgu..................       4      1   seq
    ......................................................................ucggugggacAuucguucgu..................       1      1   seq
    ......................................................................ucggugggacuuucguucgC..................       2      1   seq
    ......................................................................ucggugggacuuucguAcgu..................       1      1   seq
    ......................................................................ucggugggacuuucguuUgu..................       3      1   seq
    ......................................................................ucggGgggacuuucguucgu..................       1      1   seq
    ......................................................................ucggugggacuuucguuAgu..................       1      1   seq
    ......................................................................uAggugggacuuucguucgu..................       2      1   seq
    ......................................................................uUggugggacuuucguucgu..................       3      1   seq
    ......................................................................ucggugggacuCucguucgu..................       1      1   seq
    ......................................................................ucggugggacuuucguucgG..................       6      1   seq
    ......................................................................ucggugggacuuucgAucgu..................       4      1   seq
    ......................................................................ucggugggaAuuucguucgu..................       1      1   seq
```

**Star** **Mature**

```
uguacgcccgucgcccacgagcgauuguuucucgugaucacgugucuuuguaucaauacaaaauggcggaucggugggacuuucguucguucggggcgggcggauuaa
......................................................................Acggugggacuuucguucgu..................   9       1    seq
......................................................................ucAgugggacuuucguucgu..................   3       1    seq
......................................................................ucggugggacuuuGguucgu..................   1       1    seq
......................................................................ucgguggAacuuucguucgu..................   2       1    seq
......................................................................ucggAgggacuuucguucgu..................   1       1    seq
......................................................................ucggugggacuuucguucCu..................   1       1    seq
......................................................................ucggugggacuuuUguucgu..................   5       1    seq
......................................................................ucgAugggacuuucguucgu..................   2       1    seq
......................................................................ucggugggacuAucguucgu..................   1       1    seq
......................................................................ucggugggacuuucguucgA..................   5       1    seq
......................................................................ucgguAggacuuucguucgu..................   3       1    seq
......................................................................ucggugggacuuCcguucgu..................   1       1    seq
......................................................................Gcggugggacuuucguucgu..................   4       1    seq
......................................................................ucggugAgacuuucguucgu..................   7       1    seq
......................................................................ucggugggacuuucguucgu..................   2827    0    seq
......................................................................ucggugggacuuAcguucgu..................   1       1    seq
......................................................................ucgguAggacuuucguucguu.................   79      1    seq
......................................................................ucggugggacuuucguucUuu.................   2       1    seq
......................................................................ucggugggacuuucguucgGu.................   7       1    seq
......................................................................ucggugggacuuucguucAuu.................   5       1    seq
......................................................................ucggugggacuuucguAcguu.................   12      1    seq
......................................................................ucggugggaGuuucguucguu.................   1       1    seq
......................................................................ucAgugggacuuucguucguu.................   64      1    seq
......................................................................ucggugggacuuucguuUguu.................   38      1    seq
......................................................................ucggugggacuuucgAucguu.................   35      1    seq
......................................................................ucggugggacCuucguucguu.................   4       1    seq
......................................................................ucggugggacuuucgGucguu.................   3       1    seq
......................................................................uUggugggacuuucguucguu.................   70      1    seq
......................................................................ucggugggacuuucguucguA.................   12      1    seq
......................................................................ucggugggacuuucguuGguu.................   1       1    seq
......................................................................Gcggugggacuuucguucguu.................   53      1    seq
......................................................................ucggugggacuuuUguucguu.................   74      1    seq
......................................................................ucggugggacuuucguucCuu.................   1       1    seq
......................................................................ucggugggacuuGcguucguu.................   2       1    seq
......................................................................ucggugggacuuucguucgCu.................   3       1    seq
......................................................................uNggugggacuuucguucguu.................   1       1    seq
......................................................................ucggugggacuAucguucguu.................   16      1    seq
......................................................................ucgguUggacuuucguucguu.................   3       1    seq
......................................................................ucggugggacuuCcguucguu.................   7       1    seq
......................................................................ucggugggUcuuucguucguu.................   4       1    seq
......................................................................ucggugggacuuucUuucguu.................   1       1    seq
......................................................................ucggugggacuuucguucguG.................   13      1    seq
......................................................................ucggugggacuuucAuucguu.................   3       1    seq
......................................................................ucggugggacuuucguucgAu.................   11      1    seq
......................................................................Ncggugggacuuucguucguu.................   4       1    seq
......................................................................ucggugggCcuuucguucguu.................   1       1    seq
......................................................................ucggugggacAuucguucguu.................   6       1    seq
......................................................................ucgguggCacuuucguucguu.................   1       1    seq
......................................................................ucggugggacuuucguuAguu.................   10      1    seq
......................................................................Ccggugggacuuucguucguu.................   6       1    seq
......................................................................ucggugCgacuuucguucguu.................   5       1    seq
......................................................................ucgguggAacuuucguucguu.................   8       1    seq
......................................................................ucggugggaAuuucguucguu.................   12      1    seq
......................................................................ucggugggacuuuAguucguu.................   11      1    seq
......................................................................ucggAgggacuuucguucguu.................   36      1    seq
......................................................................ucggugAgacuuucguucguu.................   50      1    seq
......................................................................ucggugggacuuucguucguu.................   37632   0    seq
......................................................................uAggugggacuuucguucguu.................   16      1    seq
......................................................................ucggugUgacuuucguucguu.................   13      1    seq
......................................................................ucggugggacuuucguCcguu.................   6       1    seq
......................................................................ucgAugggacuuucguucguu.................   7       1    seq
......................................................................ucggugggacuuAcguucguu.................   10      1    seq
......................................................................ucggugggacuGucguucguu.................   1       1    seq
......................................................................ucggugggaUuuucguucguu.................   4       1    seq
......................................................................ucggugggacuuucguucguC.................   1       1    seq
......................................................................Acggugggacuuucguucguu.................   86      1    seq
......................................................................ucggugggGcuuucguucguu.................   6       1    seq
......................................................................ucUgugggacuuucguucguu.................   4       1    seq
......................................................................ucggugggacuuucgCucguu.................   5       1    seq
......................................................................ucggCgggacuuucguucguu.................   8       1    seq
```

# Star Mature

```
uguacgcccgucgcccacgagcgauuguuucucgugaucacgugucuuuguaucaauacaaaauggcggaucggugggacuuucguucguucggggcgggcggauuaa
......................................................................ucggugggacuuuGguucguu.................  7      1  seq
......................................................................ucgguggacuCucguucguu.................  5      1  seq
......................................................................ucCgugggacuuucguucguu.................  5      1  seq
......................................................................ucgguggUacuuucguucguu.................  2      1  seq
......................................................................ucgUugggacuuucguucguu.................  4      1  seq
......................................................................ucggugggacGuucguucguu.................  2      1  seq
......................................................................ucggugggacuuucguGcguu.................  1      1  seq
......................................................................uGggugggacuuucguucguu.................  1      1  seq
......................................................................ucggugggacuuucguuGguuc................  6      1  seq
......................................................................ucggugggacuuucguucguuG................  10     1  seq
......................................................................ucggugggacuuucguAcguuc................  35     1  seq
......................................................................ucggGgggacuuucguucguuc................  6      1  seq
......................................................................ucggugggacuuuGguucguuc................  4      1  seq
......................................................................uAggugggacuuucguucguuc................  32     1  seq
......................................................................ucggugggacuuucguGcguuc................  1      1  seq
......................................................................ucggugggacuuAcguucguuc................  30     1  seq
......................................................................ucggAgggacuuucguucguuc................  82     1  seq
......................................................................ucgguggga Uuuucguucguuc...............  37     1  seq
......................................................................ucggugggacuuucguuAguuc................  15     1  seq
......................................................................ucgCugggacuuucguucguuc................  2      1  seq
......................................................................ucggugggacuuucUuucguuc................  3      1  seq
......................................................................ucggugggacuuucguucguAc................  24     1  seq
......................................................................ucggugggacuuucgCucguuc................  11     1  seq
......................................................................ucggugggacCuucguucguuc................  13     1  seq
......................................................................ucggugggacuuucguucgGuc................  1      1  seq
......................................................................ucggugggacuuucguCcguuc................  6      1  seq
......................................................................ucggugggacuuucgAucguuc................  69     1  seq
......................................................................ucggugAgacuuucguucguuc................  206    1  seq
......................................................................ucggugggacuuucAuucguuc................  17     1  seq
......................................................................ucgAugggacuuucguucguuc................  9      1  seq
......................................................................ucggugggacuuucguucguCc................  9      1  seq
......................................................................ucggCgggacuuucguucguuc................  12     1  seq
......................................................................ucggugggacuuCcguucguuc................  12     1  seq
......................................................................ucggugggCcuuucguucguuc................  8      1  seq
......................................................................ucggugggacuuucguucCuuc................  7      1  seq
......................................................................ucggugggacuCucguucguuc................  15     1  seq
......................................................................ucUgugggacuuucguucguuc................  12     1  seq
......................................................................ucggugggacuuuCCuucguuc................  3      1  seq
......................................................................ucggugggacuuucguucAuuc................  19     1  seq
......................................................................ucggugggUcuuucguucguuc................  3      1  seq
......................................................................uGggugggacuuucguucguuc................  3      1  seq
......................................................................ucggugCgacuuucguucguuc................  4      1  seq
......................................................................ucgguUggacuuucguucguuc................  7      1  seq
......................................................................Ncggugggacuuucguucguuc................  15     1  seq
......................................................................ucggugggacuuucguuUguuc................  57     1  seq
......................................................................ucggugggacuuucguucgCuc................  16     1  seq
......................................................................ucgguCggacuuucguucguuc................  9      1  seq
......................................................................uUggugggacuuucguucguuc................  181    1  seq
......................................................................ucgUugggacuuucguucguuc................  5      1  seq
......................................................................ucggugggacuuucguucguGc................  2      1  seq
......................................................................ucAgugggacuuucguucguuc................  241    1  seq
......................................................................ucggugggacuuucguucguuA................  299    1  seq
......................................................................Gcggugggacuuucguucguuc................  171    1  seq
......................................................................ucggugggacuuGcguucguuc................  1      1  seq
......................................................................ucggugggacuuuUguucguuc................  117    1  seq
......................................................................ucggugUgacuuucguucguuc................  23     1  seq
......................................................................ucggugggacuuuAguucguuc................  20     1  seq
......................................................................ucgguAggacuuucguucguuc................  338    1  seq
......................................................................Ccggugggacuuucguucguuc................  17     1  seq
......................................................................ucggugggacuuucguucguuU................  8996   1  seq
......................................................................ucgguggga Auuucguucguuc...............  26     1  seq
......................................................................ucgguggAacuuucguucguuc................  22     1  seq
......................................................................ucggugggacuAucguucguuc................  42     1  seq
......................................................................ucgguggCacuuucguucguuc................  8      1  seq
......................................................................ucggugggacuuucguucUuuc................  3      1  seq
......................................................................ucgguggUacuuucguucguuc................  3      1  seq
......................................................................ucggugggacAuucguucguuc................  22     1  seq
......................................................................ucggugggacGuucguucguuc................  8      1  seq
......................................................................ucggugggacuuucguucgAuc................  37     1  seq
......................................................................ucggugggacuuucguucguuc................  82559  0  seq
```

```
                Star                                                  Mature
uguacgcccgucgcccacgagcgauuguuucucgugaucacgugucuuuguaucaauacaaaauggcggaucggugggacuuucguucguucggggcgggcggauuaa
......................................................................ucCgugggacuuucguucguuc................   10     1     seq
......................................................................ucggugggacuGucguucguuc................   3      1     seq
......................................................................ucggugggGcuuucguucguuc................   10     1     seq
......................................................................ucggugggacuuucgGucguuc................   3      1     seq
......................................................................ucggugggaGuuucguucguuc................   4      1     seq
......................................................................Acggugggacuuucguucguuc................   196    1     seq
......................................................................ucggugggacuuucguucguucC...............   47     1     seq
......................................................................ucggugggacuuucguucguucU...............   2931   1     seq
......................................................................ucggugggacuuucguucguucA...............   3307   1     seq
......................................................................ucggugggacuuucguucguuUg...............   2      1     seq
......................................................................ucggugggacuuucguucguucg...............   13     0     seq
......................................................................ucggugggacuuucguucguucAg..............   3      1     seq
.......................................................................cggugggacuuucguucguu.................   1      0     seq
.......................................................................Uggugggacuuucguucguu.................   1      1     seq
.......................................................................cggugggacuuucguucguuc................   10     0     seq
.......................................................................Uggugggacuuucguucguuc................   1      1     seq
.......................................................................cggugggacuuucguucguucU...............   2      1     seq
........................................................................gguAggacuuucguucguu.................   1      1     seq
........................................................................ggugggacuuucguucguu.................   6      0     seq
........................................................................ggugggacuuucguucguuc................   18     0     seq
........................................................................ggugggacuuucguucguuU................   3      1     seq
........................................................................ggugggacuuucguucguucA...............   2      1     seq
.........................................................................gugggacuuucguucguuc................   1      0     seq
```
